# Biodegradability of Amniotic Membrane as Potential Scaffold for Periodontal Regeneration

**DOI:** 10.7759/cureus.45394

**Published:** 2023-09-17

**Authors:** Kung Ee Ling, Siti Mardhiah Roslan, Haslina Taib, Zurairah Berahim

**Affiliations:** 1 Dental Clinic, School of Dental Sciences, Universiti Sains Malaysia, Kota Bharu, MYS; 2 Unit of Periodontics, School of Dental Sciences, Universiti Sains Malaysia, Kota Bharu, MYS

**Keywords:** barrier membrane, periodontal regeneration, scaffold, degradation rate, amniotic membrane

## Abstract

Background

In the periodontal regenerative procedure, the membrane used should possess good mechanical stability with suitable resorption time to allow restoration of the lost periodontium. Amniotic membrane (AM) has regenerative potential as a scaffold or barrier membrane due to its various beneficial properties. However, its degradation rate is not clearly reported.

Methodology

This study aimed to evaluate the resorption capacity of AM and its surface architecture after being subjected to hydrolytic degradation analysis in phosphate buffer solution (PBS). AM was cut into sizes of 10 × 10 mm^2^ for three replicates. The membranes were weighed before and at different time intervals (days 7, 14, 21, and 28) after immersion in PBS. The degradation rate was determined by the percentage of mean weight loss from the initial weight at different time intervals. The AM surface profile was observed under scanning electron microscopy (SEM) before and after 28 days of immersion.

Results

The result shows a 92% loss of weight over 28 days with the highest attained in the first seven days (67%), followed by 7%, 17%, and 1% after days 14, 21, and 28, respectively. SEM of the AM surface before the degradation test showed a polygonal shape forming a well-arranged mosaic pattern covered with microvilli. At day 28, the remaining AM appears as porous surface architecture, irregularly arranged fibers, and no microvilli seen.

Conclusions

This study demonstrated that over four weeks of degradation analysis, AM was not entirely degraded but had lost some of the microstructure. The biodegradability of AM should be further evaluated to elucidate its stability within adequate time parallel with the tissue healing process in periodontal tissue regeneration.

## Introduction

Periodontitis is an inflammatory disease resulting in substantial loss of periodontal ligament and alveolar bone. The key aim of periodontal therapy is the regeneration of the tissues that have been destroyed once the inflammatory component of the disease has been controlled [1]. Periodontal regeneration is the reconstruction of a lost or damaged component to restore the form and function of injured structures. It is a multifaceted, intricate process that encompasses cell adhesion, migration, proliferation, and differentiation in an organized order [2]. Procedures for regenerating periodontal tissue include guided tissue regeneration (GTR), root biomodifications, soft tissue grafts, bone grafts, growth factors, and/or a combination of these procedures [1,3-6].

GTR is one of the regenerative procedures that utilize a barrier membrane between the soft tissue flap and root surface to prevent apical migration of oral epithelium and allow repopulation of periodontal fibroblast into the defect sites [7]. The membrane may also act as a scaffold for cell growth in tissue engineering. As a physical barrier, apart from biocompatibility, the membranes should possess adequate host response and be able to withstand the inflammatory process. These require good mechanical characteristics such as permeability, stability, elasticity, flexibility, plasticity, and the ability to resorb at a rate consistent with the replacement of damaged tissue during healing. Additionally, scaffolds should permit cell adherence and the delivery of biomodulatory substances such as growth factors and genetic material [8].

The amniotic membrane (AM) is a thin layer of the innermost side of the fetal placenta encircled by the embryo in the amniotic fluid of the amniotic cavity. It consists of three layers, namely, the epithelial layer, basement membrane, and mesenchymal layer. The epithelial layer is a single layer of epithelial cells, and the basement membrane is a thin membrane composed of reticular fibers that attach to the epithelial layer by interdigitation [9,10]. The mesenchymal layer is an avascular collagen matrix that is divided into compact, fibroblast, and spongy layers. The structure and function of AM have been studied, especially considering its pluripotent cell characteristics, which make it a desirable source for tissue transplantation. This allograft also possesses various beneficial properties that include anti-inflammatory, anti-angiogenic effects, inhibition of scar formation, and promoting epithelialization that enhance the healing process [9,11-13].

AM closely mimics the oral mucosal basement membrane and has different forms of laminins, which can enhance tissue adhesion and stimulate regeneration [14]. These properties contribute to improved healing of periodontal tissue resulting in reduced probing pocket depth and gain in clinical attachment level [15]. Many scaffolds are available in the market for periodontal applications such as for GTR at periodontal defect or for root coverage procedures in the management of gingival recession. The outcome of such procedures may be different with different scaffolds used [16]. The use of AM in periodontal application has become the preference of clinicians probably owing to its good properties, as mentioned earlier.

One of the important properties of the membrane is good mechanical stability that provides structural integrity at the regenerative site. It has been shown that resorbable membranes have unpredictable resorption times and degrees of degradation [17,18]. Some materials may degrade faster than the wound healing process compromising the stability. It was suggested that for a membrane to successfully serve its function and promote tissue regeneration, the recommended biodegradation time is within two to four weeks [2]. Upon two to three weeks of wound closure, periodontal healing/regeneration largely appears complete, to be followed by remodeling/tissue maturation to meet functional demands [19]. The biodegradation rate of AM is not clearly reported in the literature; thus, this study was conducted to elucidate the resorptive capacity of this membrane by using hydrolytic degradation analysis. The findings from this study would support the potential application of AM as a scaffold or barrier membrane in periodontal regenerative procedures.

## Materials and methods

Preparation of amniotic membrane

This is an in-vitro study using AM in glycerol preserved with a size of 50 × 50 mm^2^. AM was purchased from USM Tissue Bank, Malaysia. The membrane was removed from the solution with a sterilized tweezer and washed three times in sterile phosphate buffer solution (PBS) in a petri dish to remove the excess glycerol. AM was spread out and carefully cut into pieces measuring 10 × 10 mm^2^ using a sterile scalpel blade (No. 15, Medesy, Italy) and allowed to dry for 15 minutes before testing. The membrane was prepared in triplicates and all procedures were performed in the Craniofacial Science Laboratory at the School of Dental Sciences, Universiti Sains Malaysia. The workspace was disinfected with absolute alcohol (Sigma-Aldrich, Germany) before conducting the procedures.

Hydrolytic degradation analysis

The degradation rate of AM was assessed by a hydrolytic degradation test [20]. This test monitors the behavior of the scaffold/membranes in PBS at 37°C by observing the changes in weight and morphology of the membrane after different time periods. The baseline weight of the dry AM was measured for day zero using a digital weighing machine (A&D Weighing GR-200, Japan) with an accuracy of 0.0001 g. Subsequently, all samples were immersed in the PBS and incubated at 37°C. The membranes were retrieved on day seven and left to dry in a desiccator for 15 minutes, and the weight of all samples was measured. Later these samples were immersed again in sterile PBS and incubated at 37°C. These procedures were repeated on days 14, 21, and 28 [17] to evaluate the AM degradation process for 28 days.

The degradation rate was determined based on the percentage of the AM weight loss calculated using the following formula [20]:

Gravimetric weight loss (%) = (Wi − Wf)/Wi × 100

Wi = Initial weight of the membrane before incubating in PBS.

Wf = Final weight of the membrane after each day of assessment (7, 14, 21, and 28 days).

The gravimetric weight loss on each day of assessment for all samples was tabulated and the data are presented as a chart of the degradation profile.

Analysis of amniotic membrane surface architecture

One sample of AM for day zero and day 28 was retrieved for scanning electron microscopy (SEM) analysis to observe any changes in the surface architecture before and after the 28-day degradation test. The membranes were fixed using 8% formaldehyde at 4°C for 48 hours. After that, it was washed with PBS and dehydrated in a series of graded alcohol solutions from 30%, 50%, 60%, 70%, 80%, 90%, and 100% for 10 minutes each. Then, the samples were soaked in hexamethyldisilazane (Sigma, USA) for 10 minutes and air-dried. The samples were mounted on suitable size plastic microscope slides before viewing under 1,000× and 5,000× magnification of SEM [21].

## Results

Amniotic membrane degradation rate

**Figure 1:**
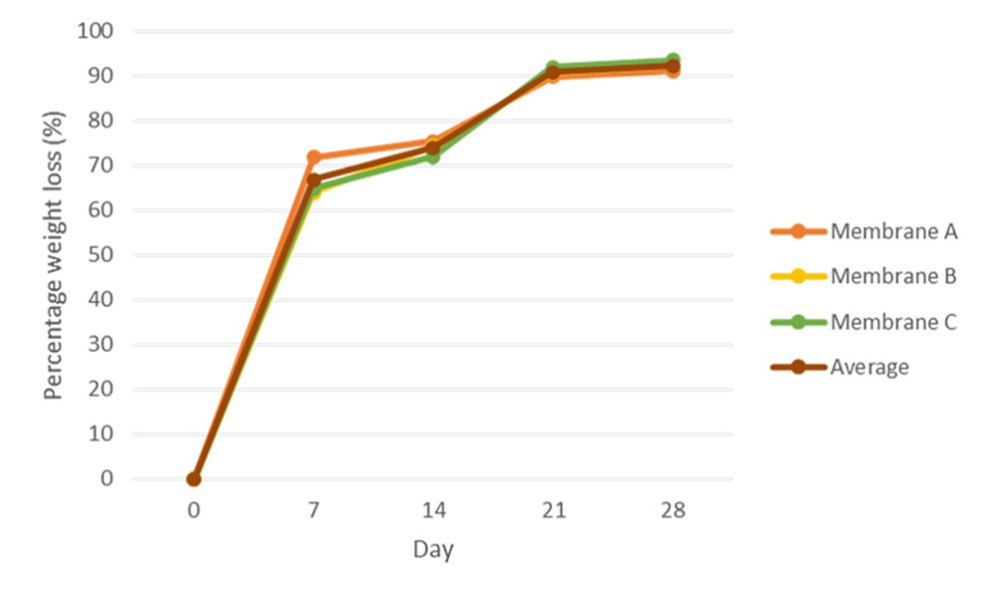
The degradation profile of amniotic membrane based on the percentage of weight loss on different days of the hydrolytic degradation test. The membrane was prepared in triplicates with the sizes of 10 × 10 mm^2^ (membranes A, B, and C).

**Figure 2:**
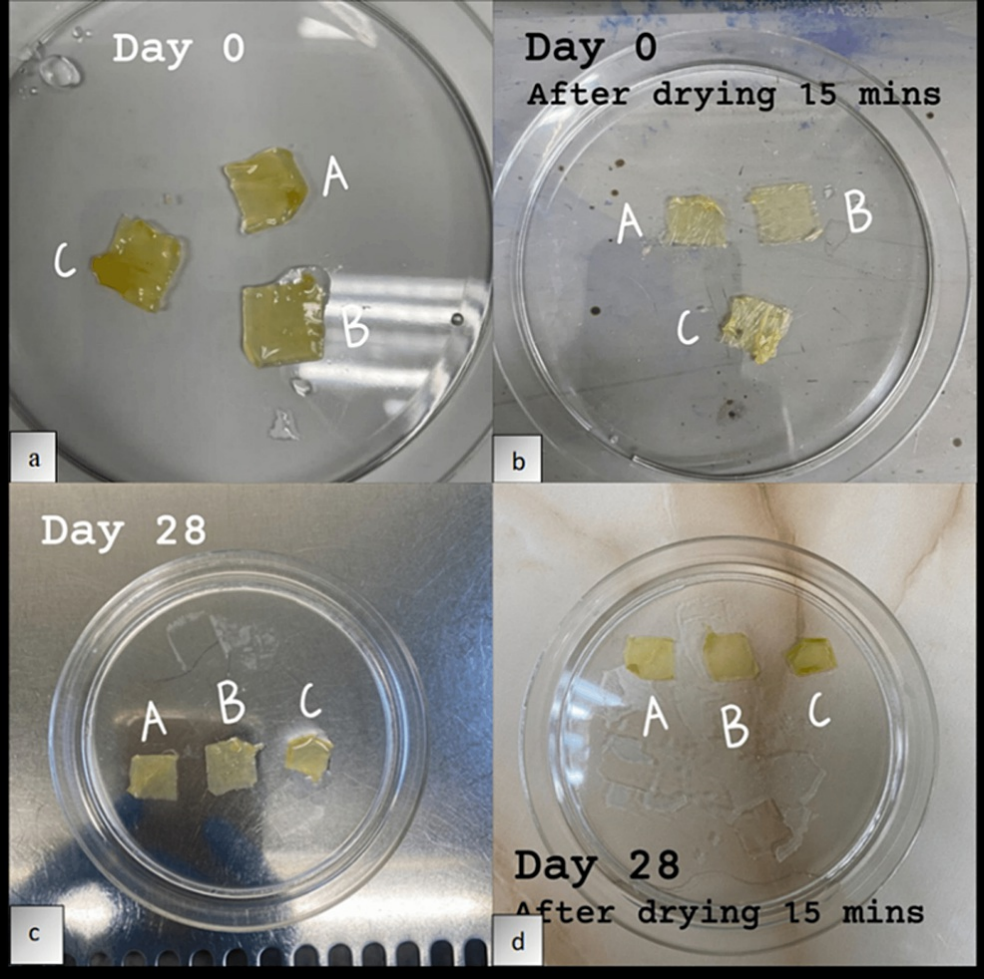
Preparation of the amniotic membrane for degradation analysis. The three pieces of amniotic membrane (A, B, and C) measuring 10 × 10 mm^2^ in a petri dish. (a) Membranes were immersed in PBS on day zero before the hydrolytic degradation test. (b) Membranes were dried for 15 minutes on day zero. (c) Membranes were immersed in PBS on day 28 after the hydrolytic degradation test and (d) dried for 15 minutes before weight measurement.

Figure 1 shows the degradation rate profile of AM measured at day zero, seven, 14, 21, and 28 for the triplicates at different time intervals. All three membranes (membranes A, B, and C) showed a similar pattern of weight loss from day zero to day 28. The slope of the line is very steep from day zero to day seven representing the highest percentage of weight loss, reducing about 67% from the initial weight. From day seven to day 14, the membranes showed an additional 7% weight loss followed by 17% and 1% on day 21 and 28, respectively. Overall, the membranes showed about 92% percentage of weight loss after 28 days of immersion in PBS. With the remaining 8% of weight, the AM still appeared intact physically, but with mild shrinkage in size after 28 days (Figure 2).

Scanning electron microscopy analysis of amniotic membrane surface architecture before and after the hydrolytic degradation test

**Figure 3:**
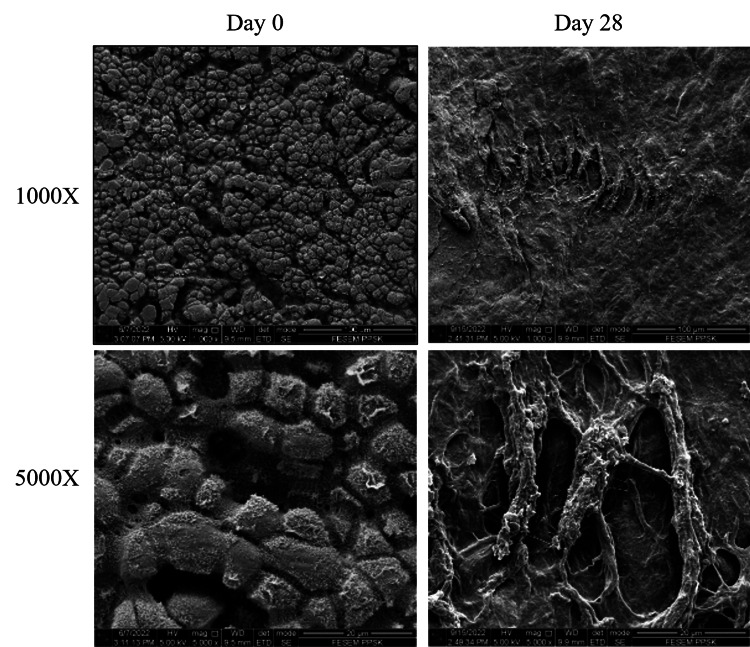
Scanning electron microscopy analysis of the amniotic membrane (AM) before and after 28 days of hydrolytic degradation test at 1,000× and 5,000× magnifications. The well-arranged mosaic pattern and abundant fibers of AM seen on day zero appeared as more porous with less dense irregularly arranged on day 28 indicate the destruction of the original structures.

On day zero, the AM surface appeared as a well-arranged mosaic pattern with compact polygonal fiber bundles arrangement covered with microvilli. There were abundant fibers in an almost homogeneous pattern. Figure 3 shows the surface profile of AM viewed under 1,000× and 5,000× magnifications in SEM for day zero and day 28 before and after the hydrolytic test, respectively. After 28 days of immersion in PBS, the remaining AM appeared as irregularly arranged fibers and loss of microvilli. The fibers are thin and less dense compared to day zero. The surface architecture changed to more hollow and porous reflecting the loss of original structures due to the degradation process.

## Discussion

Periodontal regeneration through GTR procedures employed several biocompatible membranes as a barrier for epithelial cell exclusion from reaching the root surface, thus allowing the repopulation of periodontal ligament and bone cells into the defect sites [18]. There are various choices of membranes in the market characterized as non-resorbable or resorbable including synthetic or naturally derived from human or animal tissues [6,7,17]. The non-resorbable membrane requires a second surgical intervention that induces patient morbidity. On the other hand, resorbable membranes should ideally be degraded in a time frame that is compatible with periodontal tissue healing and regeneration.

The biodegradable property of a scaffold or barrier membrane will influence the surgical outcome of regenerative procedures such as in GTR [22]. To enhance the regeneration of periodontal tissue, membranes should typically be stable for at least four to six weeks at the wound site [23,24]. A membrane with a rapid degradation rate may easily collapse earlier than the tissue healing time. Meanwhile, prolonged degradation may hinder the formation of new tissue and may lead to poor or unsuccessful tissue restoration [24]. The results of this study found that AM underwent a degradation process in PBS by abruptly losing its weight by about 67% from day zero to day seven and continuously decreased to about 92% throughout the 28-day hydrolysis test. From direct observation, despite retaining approximately 8% of weight after the end of the analysis, AM remained physically intact with mild shrinkage in size. Based on the SEM image at baseline, AM appeared as compact collagen fibers that were well-arranged in a mosaic pattern. Collagen fibers are one of the main components of the extracellular matrix that provides a mechanical and biochemical platform for cell attachment, proliferation, as well as for blood clot formation during the tissue healing process [10,12]. Upon immersion in PBS, these fibers became thin, less dense, and lost structural integrity after 28 testing days, indicating most of the AM microstructures were degraded.

In normal conditions, the healing process takes about three weeks from the hemostasis phase to the remodeling phase, and another one to two weeks to reach maturation [2,25]. It can be assumed that the degradation rate of AM shown in this study appears to be parallel with the time taken for the complete wound healing process. Thus, AM could be a suitable barrier membrane for the proposed timeframe of tissue healing to serve its function. However, it is essential to note that in the oral cavity, several factors, including membrane exposure, oral bacterial colonization, and differences in the composition, topologies, and qualities of the membrane itself, may alter the degradation rate of the membrane utilized in GTR procedures. The ability to manage the degradation of the scaffold is critical. It was initially believed in tissue engineering that the scaffolds would disintegrate and resorb as the tissue grows. It is crucial to consider that the ingrowth and maturation of different tissues occur at various time points [22].

A scaffold that is suitable for biomedical applications must be highly porous, interconnected, and have a fibrous structure that supports biological processes, including cell growth, migration, and differentiation [16]. Previous in-vitro studies [10,26] demonstrated that fibroblast cells are able to adhere to collagen membrane for seven days; hence, the membrane needs to be intact and remain stable within the same duration after the surgical procedure to allow the establishment of the periodontal ligament cell attachment and proliferation [18]. This study demonstrated that there are significant changes in AM microstructures after 28 days of hydrolytic degradation test. Although it remained physically intact, the mechanical strength may already be affected which could reduce the stability [9]. In clinical application, the membrane should be able to preserve its mechanical characteristics and possess structural integrity to withstand mechanical interference both during the regeneration process and during routine oral activities, thus requiring longer stability and an appropriate biodegradation rate [24,27].

Hence, to be applied clinically, modification of AM structures was suggested to increase its tensile strength which may prolong its degradation rate. Some methods were suggested, for instance, by cross-linking AM with composite materials, physical cross-linking, and chemical cross-linking with glutaraldehyde, carbodiimide, genipin, and aluminum sulfate [9,28]. An animal study by Zhang et al. used fresh AM treated with ultraviolet cross-linking for conjunctival transplantation on rabbits and compared it with control AM [29]. It was also noted that the glutaraldehyde-treated AM was virtually completely resistant to the enzymatic digestion test, while fresh and cryopreserved AM were dissolved completely by day seven. This suggests that collagen cross-linking using glutaraldehyde leads to a significant increase in the biomechanical strength and enzymatic resistance of AM, which can be employed to prolong the degradation time of AM in the oral cavity [9,29]. Thus, it can be proposed that the placement of AM as a barrier membrane prevents the early exposure of the surgical site to produce the desired effects [30].

On the other hand, a study by Ardakani et al. found that the viability of fibroblast cells cultured on the cross-linking membrane reduced after seven days of observation when compared with native collagen membranes [26]. It can be assumed that the cross-linking of collagen membranes may somewhat affect cell attachment despite increasing the membrane degradation time. The effectiveness of AM cross-linking for cell proliferation should, therefore, be further assessed.

Despite the findings of this study, certain limitations were encountered. The degradation of AM mostly occurs within the first seven days of the test. Even though it is not completely degraded, the ability to withstand as a barrier membrane in GTR to prevent epithelial cell downgrowth into the defect site may be compromised. This study did not evaluate the tensile or mechanical strength of AM; hence, it was unable to draw any firm conclusions. Assessment of the physical and biochemical characteristics of AM can be included in future studies to enhance the findings. Another limitation is the number of AM samples used was so small that statistical analysis for comparison of weight changes could not be applied. Apart from the hydrolytic degradation test, the biodegradation rate of AM can be tested by other analyses such as the collagenase degradation test in which the membrane resistance toward enzymatic digestion by 0.1% collagenase solution is determined [17]. Future studies should employ this test and other collagen membranes into which comparative findings could be made.

## Conclusions

Within the study limitations, AM structures were altered and showed fiber destruction upon 28 days of hydrolytic degradation test. The rate of AM degradation is likely to be appropriate to allow periodontal tissue healing. However, further studies particularly in-vivo and clinical trials are recommended to evaluate its resorptive capacity and stability as a barrier membrane before a definitive conclusion can be made.
